# Oculomotor behavior during non-visual tasks: The role of visual saliency

**DOI:** 10.1371/journal.pone.0198242

**Published:** 2018-06-22

**Authors:** Dekel Abeles, Roy Amit, Shlomit Yuval-Greenberg

**Affiliations:** 1 School of Psychological Sciences, Tel Aviv University, Tel-Aviv, Israel; 2 Sagol School of Neuroscience, Tel Aviv University, Tel-Aviv, Israel; State University of New York Downstate Medical Center, UNITED STATES

## Abstract

**Background:**

During visual exploration or free-view, gaze positioning is largely determined by the tendency to maximize visual saliency: more salient locations are more likely to be fixated. However, when visual input is completely irrelevant for performance, such as with non-visual tasks, this saliency maximization strategy may be less advantageous and potentially even disruptive for task-performance. Here, we examined whether visual saliency remains a strong driving force in determining gaze positions even in non-visual tasks. We tested three alternative hypotheses: a) That saliency is disadvantageous for non-visual tasks and therefore gaze would tend to shift away from it and towards non-salient locations; b) That saliency is irrelevant during non-visual tasks and therefore gaze would not be directed towards it but also not away-from it; c) That saliency maximization is a strong behavioral drive that would prevail even during non-visual tasks.

**Methods:**

Gaze position was monitored as participants performed visual or non-visual tasks while they were presented with complex or simple images. The effect of attentional demands was examined by comparing an easy non-visual task with a more difficult one.

**Results:**

Exploratory behavior was evident, regardless of task difficulty, even when the task was non-visual and the visual input was entirely irrelevant. The observed exploratory behaviors included a strong tendency to fixate salient locations, central fixation bias and a gradual reduction in saliency for later fixations. These exploratory behaviors were spatially similar to those of an explicit visual exploration task but they were, nevertheless, attenuated. Temporal differences were also found: in the non-visual task there were longer fixations and later first fixations than in the visual task, reflecting slower visual sampling in this task.

**Conclusion:**

We conclude that in the presence of a rich visual environment, visual exploration is evident even when there is no explicit instruction to explore. Compared to visually motivated tasks, exploration in non-visual tasks follows similar selection mechanisms, but occurs at a lower rate. This is consistent with the view that the non-visual task is the equivalent of a dual-task: it combines the instructed task with an uninstructed, perhaps even mandatory, exploratory behavior.

## Introduction

We are constantly exposed to abundant sensory input from our environment. This input is sometimes relevant, for instance when observing an object with the intention of acting upon it, but in many cases the input is entirely irrelevant for our behavior and could even be disruptive [1–3]. This is the case, for example, when performing non-sensory tasks that are driven by internal cognitive processes. Much of our time is spent performing such tasks: planning, memorizing or processing knowledge in ways that do not require interacting with our immediate external environment. In the visual modality, irrelevant input could have been easily avoided by shutting the eye-lids. Despite this, we tend to spend most of our waking hours with open eyes allowing the flow of potentially disruptive visual input.

Regardless of the task, visual input is always only partially processed. Starting from the distribution of photoreceptors across the retina and higher up throughout the visual hierarchy, there is a resolution gradient which is maximal at the center of fixation ("fovea") and minimal at the far periphery. Attentional resources are also deployed to prioritize visual input. In free-view contexts, when no specific instruction regarding eye movements is given, attentional prioritization is achieved through shifts of gaze. Fast ballistic eye movements known as "saccades" relocate the fovea from one location of interest to another. This process of attentional selection through eye movements can be driven by either top-down or bottom-up mechanisms. Top-down selection is internally driven by the subjective motivations of the observer, such as his/her current goals or expectations. Bottom-up selection, on the other hand, is driven by the external visual input and is determined by its characteristics [4–6].

The role of bottom-up selection in driving gaze shifts during visual exploration has been successfully modeled by the concept of visual saliency. Saliency mapping algorithms simulate the bottom-up prioritization processes conducted by the visual system to guide eye movements. An example for one of the earliest and most influential saliency models, which was based on a biologically plausible framework, is that of Itti, Koch and Nieburs [7]. This algorithm was followed by numerous other saliency mapping algorithms which vary in their logic and implementations, but share the same goal: to assign a saliency value for each spatial coordinate of an image to estimate the probability that this coordinate would be fixated by free-viewing observers [8–11]. Some of the newer algorithms integrated additional aspects of human vision to enhance their performance. This included the inhomogeneous retinal acuity, oculomotor biases [12] and even top-down processes [13–15]. Recently, saliency algorithms were developed by training neural networks on human fixation data to capture a wide variety of features that would enhance predictions of future gaze locations [16–18]. A possible disadvantage of this type of algorithms may be their intangibility, as the underlying features driving their computation remain non-explicit. However, for the purpose of this study we use saliency merely as predictors for gaze positions during scanning, regardless of their physiological validity. We, therefore, chose to use one of the best currently available algorithms, which is based on neural networks and its gaze predictions are publicly shared [16].

Testing the match between saliency models and fixation maps requires that participants perform gaze shifts on the examined images. This was achieved either by explicit visual exploration tasks, such as scene memorization [19–21] or visual search [19,22–24]; or by the less explicit “free-viewing” task where participants are presented with images without any instructions other than to look at them [15,25–29].

With these visual tasks, saliency maximization is one of the critical factors in determining gaze positioning. However, during non-visual tasks, such as answering arithmetic or biographical questions, irrelevant visual information may impair task performance. For example, eye-witnesses were found to provide more accurate testimonies when they were instructed to close their eyes [30,31]. There is also a known tendency to avert the gaze away from visual stimulation during non-visual tasks [32–34] and known behavioral costs for not doing so [30,31,35–39]. In a recent study, we demonstrated the link between gaze aversion and motion saliency by showing that participants tend to avert their gaze for longer durations when a task-irrelevant dynamic visual stimulus was moving fast rather than slow [40].

It could therefore be hypothesized that following a saliency maximization criterion during non-visual task would be disadvantageous for task performance. In the present research we examined the role of visual saliency during a non-visual task, for which visual information was entirely irrelevant. We tested three alternative hypotheses: a) That saliency is disadvantageous for non-visual tasks and would therefore be processed and used to steer the gaze away from potential visual distractions; b) That saliency information is unnecessary during non-visual tasks and would therefore not be processed or acted upon once the visual modality is irrelevant; c) That saliency maximization is mandatory and would determine gaze positions even during non-visual tasks. These three hypotheses predict different experimental outcomes: the first predicts a negative correlation between gaze positioning and saliency, reflecting a saliency minimization process; the second predicts no correlation between saliency and gaze positioning; and the third predicts that gaze positions would be positively correlated with saliency, as found during visual tasks.

To test these competing hypotheses, we performed two eye-tracking experiments. In the first experiment, we examined scan patterns and gaze related statistics, in participants who were presented with complex images while being engaged in either a non-visual (continuous mental arithmetic) or a visual exploration task. In the second experiment we examined scan patterns while participants were presented with simple non-semantic dynamic stimuli, which had binary salient values (each image location was either salient or non-salient). This experiment was designed to test the role of visual saliency free of saliency algorithms and semantic information.

## Methods

### Experiment 1

#### Participants

This experiment included 21 participants who participated for monetary compensation or course credit (13 females, Mean age = 23.90, Range 19–35, SD = 3.76). One participant was removed from the analysis due to low-quality eye tracking. All participants reported being healthy, with no history of neurological disorders and with normal or corrected-to-normal vision and normal hearing. The Tel Aviv University and the School of Psychology ethics committees approved the experimental protocol and the experimental sessions were conducted with the written consent of each participant.

#### Apparatus

Participants were seated in a dimly-lit room with their head supported by a chin and a forehead rest at a viewing distance of 1 m from a 24-inch LCD monitor (ASUS VG248QE) with 120Hz refresh rate and a resolution of 1920 x 1080, covering 30° of the observer's visual field. The experiments were programed in Matlab (MathWorks) using the Psychophysics Toolbox [41,42] and data were analyzed using Matlab and IBM SPSS.

Previous studies suggested that observers tend to avert their gaze, away from task-irrelevant salient stimuli [32,35,40]. To minimize the tendency of observers to look away from the screen during non-visual tasks, we added a white cardboard tunnel, blocking the field-of-view outside of the computer's screen borders. Participants were still able to shift their gaze toward the tunnel walls, but the tunnel acted as a strong cue to keep the gaze at the screen despite the absence of any explicit instructions to do so.

Binocular gaze position was monitored using infrared video-oculographic system (Eyelink 1000 Plus, SR Research) with a spatial resolution smaller than 0.01° and average accuracy of 0.25–0.5° when using a head-rest (as reported by the manufacturer), sampled at 1000Hz. Raw gaze positions were converted into degrees of visual angle based on a 9-point calibration performed at the beginning of each experimental block (on mid-gray background) and repeated when necessary.

#### Stimuli

All written text stimuli (numbers, fixation and mathematical operators) were presented in font "courier new" size 50pt. Each character in those settings covered approximately 0.66 x 1.18 visual degrees (horizontal x vertical).

The stimulus set consisted of 90 color images chosen from the MIT data set of 1003 photos [11]. Only images with a resolution of 768*1024 and with no predominant textual elements were considered for inclusion. Out of the remaining images, we chose to include images in which saliency was a good predictor of gaze position, according to the following procedure: First, a saliency map was computed for each image using the adaptive saliency whitening algorithm [23]; Second, images were sorted according to the computed area under curve (AUC) of the receiver operant characteristic curve (ROC) based on the corresponding fixation data provided with the dataset; Third, the first 90 images of this sorted list were chosen. Additionally, 25 other images were chosen randomly to be used in the practice and the test blocks (see below). The presented size of each image was 17 X 22.76 (vertical x horizontal) visual degrees. Images were presented on a mid-gray background. The chosen images were randomly assigned to two sets (45 images per set), each presented during either the mental arithmetic blocks or the visual exploration blocks, counterbalanced between participants. The order of presentation was random and none of the images appeared more than once during a session.

#### Procedure

Each experimental session included 8 blocks. One practice block (20 trials) followed by three experimental blocks (15 trials each) of the mental arithmetic condition (see below) and three visual exploration blocks (15 trials each, with no practice) followed by one recognition test block (10 trials).

**Fig 1:**
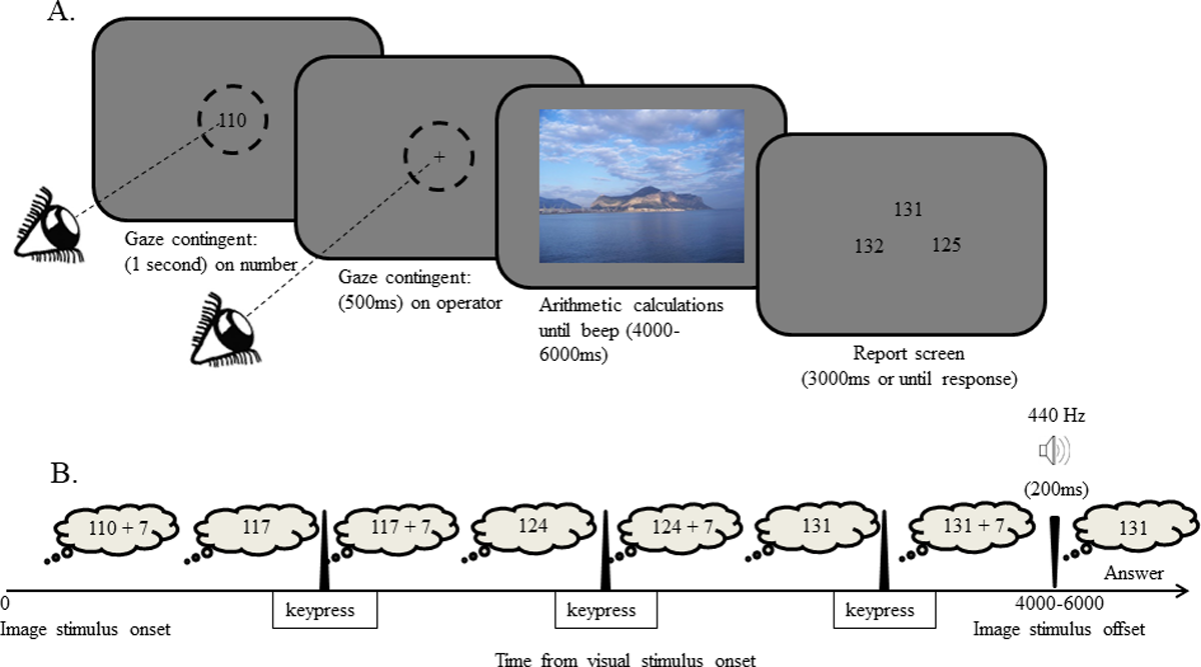
Trial procedure of the mental arithmetic task of Experiment 1. A) Schematics of the trial procedure in the mental arithmetic task. The dashed circles represent the area defined as gaze-on-target for the gaze contingent procedure (radius = 2°). The starting number in this case is 110 and the participant is requested to add 7 in each operation. The presented report screen is an example for a possible report screen following three operations. The correct answer is 131 and therefore the expected response is an upward arrow. B) Schematic representation of the mental and motor processes during the image presentation interval. In this example the participant managed to finish three operations of addition to the "starting number" and should therefore choose the "up" arrow according to its position on the report screen.

Each trial of the mental arithmetic condition began with the presentation of a 3-digit number (101–999) in the center of the screen (termed “starting number”). In a supplementary control experiment the starting-number was presented at various peripheral locations rather than at the center and the same qualitative results were found (S2 File, Control Experiment 1A). The starting numbers were the same for all participants but appeared in a random order (none was repeated more than once per session). Participants were instructed to gaze at the number, which disappeared after they did so continuously for 1 second (verified by a gaze contingent procedure). The starting number was replaced by an arithmetic operator at the same location: either a addition or a subtraction sign with equal probabilities. The operator remained on screen until the participants gazed at it for 500ms. Once the operator disappeared, an image was presented for a random duration of 4–6 seconds in most trials and 1–2 seconds in two random trials per block (termed “fast trials”). Concurrently with the offset of the image, a beep (440Hz, 200ms) was sounded to signal the beginning of the report interval, and three numbers were presented at 2° eccentricities from the center: left, up and right of center. These three numbers constituted the multiple-choice options for report (termed “report screen”). The report screen remained until a response was detected, or the acceptable response duration has passed (3 seconds). In total, each trial lasted between 5.5 and 7.5 seconds plus the time to comply with the gaze contingent instructions and the reaction times. The trial procedure of the mental arithmetic condition of Experiment 1 is depicted in **Fig 1A**.

The purpose of the mental arithmetic task was to engage participants with continuous arithmetic operations throughout the image presentation period. As soon as the operator disappeared, participants were instructed to start a continuous process of mentally subtracting or adding the number 7, depending on whether a subtraction or an addition operator was presented in that trial. They were instructed to begin with the starting number, add/subtract 7 to it and then keep repeating the same ±7 operation on each calculation result, until the beep was sounded. They were required to report the completion of each such ±7 mental operation by pressing the space bar once (but did not report the outcome of the calculation until the report period). For example, if the starting number is 110 and the operator is addition, a participant would mentally calculate 110+7 = 117 and press the space bar, then he/she would calculate 117+7 = 124 and press the space bar again and do this repeatedly for a few times. As soon as the beep was sounded and the report interval started, the participant was instructed to report the last outcome of this repeated mental calculation, by pressing the appropriate keyboard arrow key.

The correct answer (calculated by multiplying the number of space bar presses by 7 and adding/subtracting this value from the starting number) was presented as one of the multiple-choice options in 75% of the trials (25% per location). In the remaining 25% of the trials, none of the three numbers represented the correct answer, and a correct response would be to press the down arrow to indicate that the correct result was absent. The incorrect alternatives were randomly drawn from the interval of -7 to +7 (excluding 0) from the correct answer. The response was followed by a feedback sound (correct - 440Hz, incorrect - 220Hz) allowing the participants to estimate their performance in a per trial basis and improve it if necessary. The report interval was limited to three seconds, to encourage fast responses and minimize the probability that participants would perform calculation during the report interval rather than continuously throughout the trial. Trials were classified as “incorrect” when the spacebar was not pressed during the stimulus presentation, the selected answer was wrong or there was no response during the report period. At the end of each block participants were presented with their cumulative score in the task, calculated as the total number of spacebar clicks on correct trials. To encourage high performance on the task, participants were told before the experiment that their cumulative performance will determine their chances of winning a monetary prize of 200 NIS in a lottery, which took place at the end of data collection (see details below). The probability for a participant to win this lottery procedure was calculated by dividing the individual cumulative performance score by the sum of the cumulative scores of the entire sample of participants.

Instructions regarding gaze position were minimal. Participants were told that once they looked at the number, it will automatically be replaced by the arithmetic operator and that once they looked at the operator it will disappear and they should immediately start the mental calculation. They were also told that the presented images are irrelevant to this task and that they are presented simply as a mean for creating comparable lighting conditions for the two types of tasks.

In each trial of the visual exploration blocks, an image from the alternate set of images was presented for three seconds and followed by a one second blank interval (uniform grey display). Participants were instructed to view the presented images and were told that they will later be administered a recognition test. In the recognition test block, half of the trials presented a new image (i.e. never seen before), and the other half an old image (i.e. viewed during the exploration blocks). Participants were asked to classify the images as new or old by pressing the up (new) or the down (old) arrow keys, and the images remained on screen until a response was given.

### Experiment 2

#### Participants

This experiment included 20 participants (9 females, Mean age = 24.45, Range 18–29, SD = 2.76). All participants reported being healthy, with no history of neurological disorders and with normal or corrected-to-normal vision and normal hearing. The experiment has gone through the same ethical procedure as Experiment 1.

#### Apparatus

Same as in Experiment 1.

#### Stimuli

**Fig 2:**
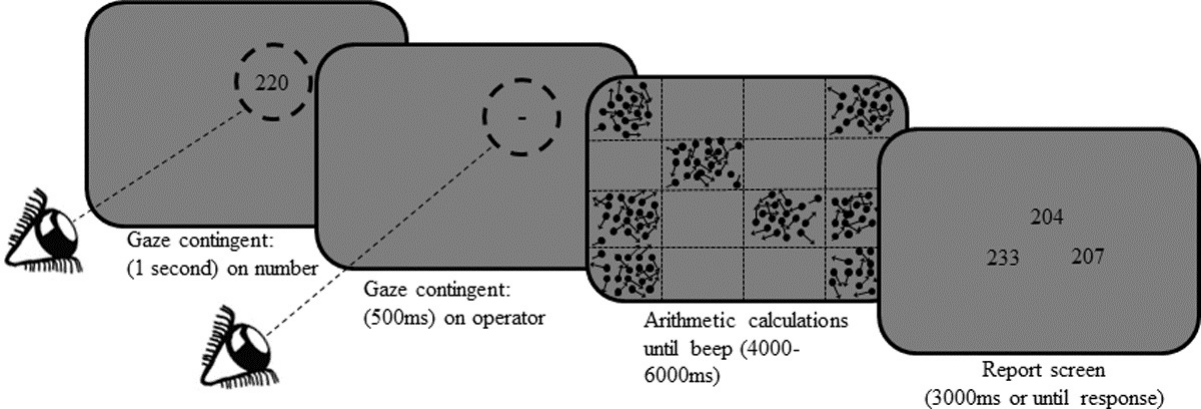
Trial procedure of the mental arithmetic task and cells-RDK stimuli. The dashed circles represent the area defined as gaze-on-target for the gaze contingent procedure (radius = 2°). The black dots with arrows represent the random dot kinematogram and the dashed horizontal and vertical lines represent the cells perimeter. The dashed lines (and arrows) are presented in the figure for illustration purposes and were not visible in the experiment’s display.

The stimuli of this experiment consisted of random dot kinematogram (RDK) with 2000 black dots (diameter 0.16° each) moving in random directions at 4.8°/sec spread across the entire screen area. Every time a dot reached the boundary of the screen its position was redrawn to be located inside the screen area. Additionally, to make the dots motion even less predictable, 100 dots per frame were randomly chosen to change their movement angle. To create areas with high and low motion saliency, the screen area was divided into a 4 by 4 rectangular grid resulting in sixteen 240 x 384 pixels rectangular cells. In 8 randomly chosen cells the RDK was visible. The other 8 cells were occluded by a background-color rectangle and appeared blank. We term this type of display “cells-RDK”. The trial procedure of Experiment 2 is depicted in **Fig 2**.

#### Procedure

This experiment included 7 blocks: one practice block (20 trials), three experimental blocks of the mental arithmetic condition (16 trials each) and three number-naming condition blocks (16 trials each). There was no test block.

The mental arithmetic blocks were identical to that of Experiment 1, except that instead of the photo images, there was a cells-RDK display (as described above). Additionally, the initial number and the operator were presented in one of four peripheral positions (up-right, up-left, down-right, down-left), at a distance of 6° from the center and not at the screen center as in experiment 1. The trial procedure of the number-naming blocks was identical to that of the arithmetic blocks except that there was no report screen. In these blocks, participants were instructed to read the starting number out-loud as soon as it appears and then wait until the next trial. An experimenter monitored responses during the experiment and verified participant's responses and compliance with the task. No calculations were required in this task.

### Eye tracking analysis (for both experiments)

#### Fixation detection

Fixations were defined as the periods between saccades and blinks. Saccades were detected using a published algorithm [43,44] with a velocity threshold of 6 standard deviations and a minimum duration parameter of 6 consecutive samples. To allow comparing our findings with previous studies in the field we included in the analysis only saccades larger than 0.5°. Smaller saccades, called microsaccades are known to have a central role on vision [45] but are not usually considered in studies on saliency and fixation maps. Blinks were detected according to the eye tracker’s built-in software. Saccades with onset-times of less than ±130ms from detected blinks were discarded from analysis. For the mental arithmetic condition, only correct trials were included. Fixations detected outside the image borders were also not included in the analysis.

#### Saliency measurements

In Experiment 1, saliency maps were calculated based on the state-of-the-art Saliency Attentive Model (SAM-ResNet; [16]), a convolutional neutral network trained on human fixation data. These maps were pre-computed and downloaded from an online repository. As a complimentary analysis, saliency maps were also computed by applying the feature-based Saliency model of Itti et al. [7] using the saliency toolbox implementation [46]. Results of this analysis are reported in the Supporting Information (S1 File).

Maps were rescaled to match the presented stimulus size and then normalized to z-scores, adjusting the average value for all pixels in each map to zero and the standard deviation to one which allows the comparison between maps. For each fixation, the average normalized saliency value was calculated for a circle with a radius of 0.5 visual degrees surrounding the fixation position on the image to account for spatial uncertainties in gaze locations. In Experiment 2, which contained artificial dynamic stimuli, saliency assessment was simplified by assigning a value of one to every pixel on the cells containing a visible RDK and minus one to pixels on the blank cells.

As a baseline for comparison of saliency values, we calculated the expected average saliency value of a random scan-path, not according to saliency information. This baseline is termed "random observer". For each participant, we constructed two sets of fixation positions to simulate two types of random observers: an unbiased observer who scans completely randomly and a biased observer who follows some general stimulus-independent oculomotor biases such as the central bias. The unbiased random observer was simulated for each participant and trial of the mental arithmetic condition (where n fixations were produced) by drawing n fixations randomly and uniformly from the entire image area. The biased observer was simulated for each participant and trial of the mental arithmetic condition (where n fixations were produced) according to the following procedure: n fixations were randomly drawn out of the entire set of all fixations in all other trials of the visual exploration (Experiment 1) or number-naming (Experiment 2) condition of that participant. For both the biased and the unbiased observers, the simulated fixations saliency was then averaged within trials and then across trials.

If visual input modulates oculomotor behavior during non-visual task, it could be expected that the spatial distribution of fixated locations would be similar across different observers when presented with the same visual stimulus. To examine this question, we computed a measurement of the similarity among participants’ scan-paths in each of the conditions (“NSS-similarity”), which is based on a measurement used by Dorr et al. [47]. This analysis was identical to the NSS analysis described above, except that the algorithm-based predictive saliency maps were replaced with empirical saliency maps which were calculated individually for each participant and condition in a leave-one-out procedure: For each image, the fixations from all other participants on the same image and condition were aggregated to create a probabilistic spatial map of fixated locations, smoothed with a Gaussian (*σ*_*x*_ = *σ*_*y*_ = 1°) and normalized to z-score.

#### Gaze away from stimulus

To estimate participants’ tendency to avert their gaze from the stimulus, we calculated the gaze-away samples proportion (GSP) as in Abeles and Yuval-Greenberg (2017). Gaze position samples were classified as Gaze-on-stimulus (0) when the gaze position was not further than 1° away from the image area or Gaze-away (1) for all other samples. The Gaze-away samples included both samples in which gaze was recorded outside the presented image area and samples in which the gaze was not recorded, typically because it was directed outside the trackable zone (due to gaze aversions or eye-lid closures). The GSPs were calculated for each trial by dividing the sum of Gaze-away samples by the number of samples in this trial within 0-3000ms relative to stimulus onset.

#### Central fixation bias

This analysis focused on the first fixations after stimulus onset, which was defined as such only if their onsets occurred at least 100ms after stimulus onset. The spatial distribution of the first fixation positions was calculated for each participant and condition. To do so, in each trial, a binary matrix with the same pixel resolution as the stimulus was created. The pixel at the first fixation coordinates was assigned a value of 1, and all other pixels were assigned the value 0. These maps were averaged across trials for each participant and condition to represent his/her individual probability to fixate at each pixel on the first fixation of a trial. These average maps were then averaged across participants, separately for each of the conditions and smoothed with a 2d Gaussian (standard deviation of 0.5°, 30 pixels).

#### Statistical analysis

Within-subject Cohen's d effect sizes were estimated using a published method [48] which adjusts the effect size according to the correlation between paired samples. Whenever the sphericity assumption was violated, the degrees of freedom were adjusted according to the Greenhouse-Geisser correction and the epsilon value is reported along with the uncorrected degrees of freedom and test statistics.

## Results

### Task compliance and behavioral performance

Accuracy-rates of the mental arithmetic task were higher than chance (0.25) for all participants in both experiments (Exp 1: *M* = 0.70, *SD* = 0.09, Range 0.55–0.84; Exp 2: *M* = 0.69, *SD* = 0.13, Range 0.42–0.92). On average, participants performed more than 2 mental operations in correct trials (Exp 1: *M* = 2.41, *SD* = 0.75, Range 1.2–3.7; Exp 2: *M* = 2.26, *SD* = 0.73, Range 1–3.9). There were significantly more mental operations in correct addition than subtraction trials (Exp 1: *Addition*: *M* = 2.75, *SD* = 1.02; *Subtraction*: *M* = 2.11, *SD* = 0.71; *t*(19) = 6.262, *p*<0.001, Cohen's d = 0.56; Exp 2: *Addition*: *M* = 2.60, *SD* = 1.03; *Subtraction*: *M* = 2.15, *SD* = 0.79; *t*(19) = 5.585, *p*<0.001, Cohen's d = 0.363). This effect cannot be explained by a trade-off between number of successful mental operations and accuracy because accuracy in addition trials was either equal to or higher than accuracy in subtraction trials (Accuracy rates: Exp 1: *Addition*: *M* = 0.74, *SD* = 0.09; *Subtraction*: *M* = 0.68, *SD* = 0.13; *t*(19) = 2.06, *p* = 0.053, *Cohen's d* = 0.54; Exp 2: *Addition*: *M* = 0.73, *SD* = 0.12; *Subtraction*: *M* = 0.67, *SD* = 0.15; *t*(19) = 2.3, *p* = 0.033, *Cohen's d* = 0.41). This indicates that the subtraction was more difficult than addition. Accuracy-rates of the memory test (classifying new/old) performed after the exploration blocks of Experiment 1 were above chance (0.5) for all participants (*M* = 0.93, *SD* = 0.097, Range 0.7–1).

### Experiment 1

#### Gaze samples away proportions (GSP)

Consistently, with previous findings on gaze aversion in non-visual tasks [32,34,35,40], we find that gaze-away samples proportions (GSPs) were significantly higher in the arithmetic task (*M* = 0.099, *SD* = 0.18) than in the exploration task (*M* = 0.016, *SD* = 0.018; t(19) = 2.21, *p* = 0.039, Cohen's d = 0.4).

#### Estimation of saliency across fixations

**Fig 3:**
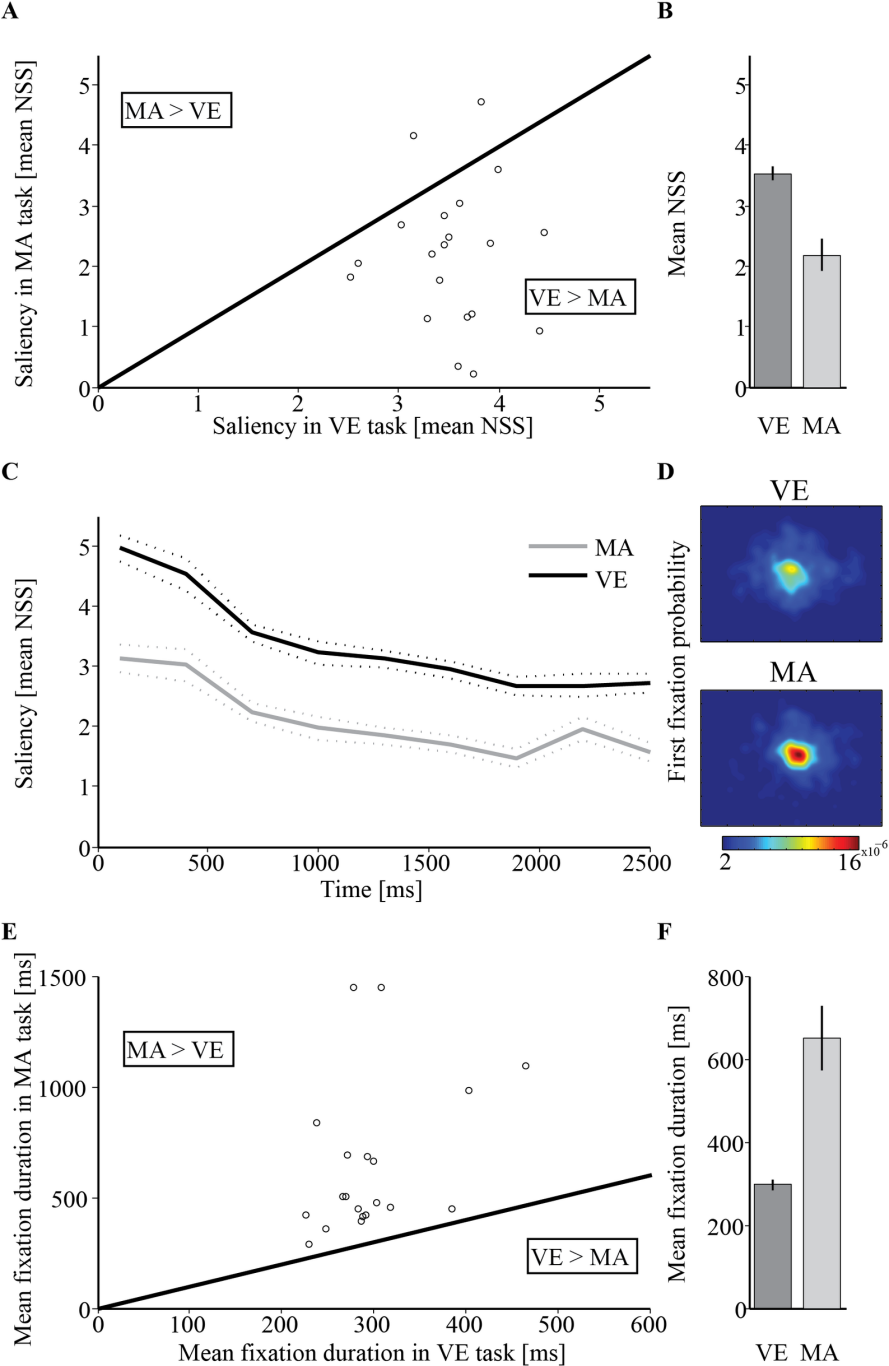
Experiment 1 results. (A) Single-subjects’ average normalized scan-path saliency (NSS) in the visual exploration (VE) condition vs. the mental arithmetic (MA) condition. Dots that are below the identity line represent participants for whom the NSS was higher in VE than in MA. (B) Grand average (N = 20) NSS per condition. Error bars denotes ±1 standard error of the mean. (C) Average NSS according to fixations onset times following image presentation (at zero). Dotted line denotes ±1 standard error of the mean. (D) Probability density maps for the spatial distribution of the first fixation occurring after image onset. (E) Single-subjects average fixation duration in the VE and MA conditions. Dots that are above the line represent participants for whom fixation duration was longer in the MA task. (F) Grand average fixation duration per condition. Error bars denotes ±1 standard error of the mean.

For each participant and condition, saliency at fixation locations was averaged across all fixations occurring during each stimulus presentation (at 100-3000ms relative to stimulus onset) to calculate the normalized scan-path saliency (NSS) which was then averaged across trials. The average NSS in both the visual exploration and the mental arithmetic condition was significantly above the expected average NSS of a random observer, both biased and unbiased (see Methods) (Arithmetic: unbiased: *M* = -0.01, *t*(19) = 8.314, *p*<0.001, *Cohen's d* = 2.64; biased: *M* = 0.46, *t*(19) = 6.225, *p*<0.001, *Cohen's d* = 2.19; Exploration: unbiased: *M* = -0.01, *t*(19) = 31.85, *p*<0.001, *Cohen's d* = 11.34; biased: *M* = 0.46, *t*(19) = 32.45, *p*<0.001, *Cohen's d* = 7.17). There was no difference in average saliency between the easier addition task and the more difficult subtraction task (*addition*: *M* = 2.22, *SD* = 1.3; *subtraction*: *M* = 2.12, *SD* = 1.24; *t*(19) = 0.441, *p* = 0.66). These results suggest that, regardless of task difficulty, visual saliency played a central role in determining fixation positions even when the task was non-visual. Nevertheless, this average saliency value was significantly higher at the visual exploration (M = 3.53, *SD* = 0.49) than at the mental arithmetic condition (M = 2.18, *SD* = 1.18; *t*(19) = 4.67, p<0.001, Cohen's d = 1.50). This indicates that saliency was more influential in determining fixation location during the visual exploration condition than while performing the mental arithmetic task. These results are depicted in **Fig 3A and 3B**.

The correspondence between saliency and fixated locations was reported to be gradually reduced from early fixations to later ones [22,25,49]. To examine this phenomenon, we divided the time interval of 100-2800ms post stimulus onset into 9 intervals of 300ms. For each trial and each time interval, we calculated the average saliency at the fixation positions where fixation onsets occurred during that time interval. Intervals in which no fixation was detected were not included in this analysis. For each participant in each viewing condition (arithmetic/exploration), these values were averaged across trials, to create one value for each time interval (9 overall) used as a dependent measure. A two-way repeated measures ANOVA was conducted on this saliency measure, with factors Time (interval 1–9, as described above) and Viewing-condition (arithmetic/exploration). A significant main effect was found for Viewing-condition (*F*(1, 19) = 23.771, *p*<0.001, *η*_*p*_^*2*^ = 0.56), reflecting higher saliency values during exploration than during arithmetic calculation. Additionally, there was a main effect of Time (*F*(8, 152) = 27.292, *p*<0.001, *η*_*p*_^*2*^ = 0.59), resulting from lower saliency for later fixations. Consistently with the previous findings, trend analysis revealed a significant negative linear trend across time (*F*(1, 19) = 133.21, *p*<0.001, η_p_^2^ = 0.87), indicating that saliency was reduced with time.

There was no evidence for an interaction between Time and Viewing-condition (*F*(8, 152) = 1.273, *p* = 0.261, *η*_*p*_^*2*^ = 0.063). We nevertheless examined the linear trends separately for the two viewing-conditions and found a significant negative linear trend for both the exploration condition (*F*(1,19) = 238.2, *p*<0.001, *η*_*p*_^*2*^ = 0.926) and the mental arithmetic condition (*F*(1,19) = 27.85, *p*<0.001, *η*_*p*_^*2*^ = 0.594; **Fig 3C**). The effect size of the linear trend of the exploration task was quantitatively stronger than that of the mental arithmetic task (*η*_*p*_^*2*^ = 0.926 vs. *η*_*p*_^*2*^ = 0.594), but this difference was not consistent enough to be validated by a significant interaction.

A separate repeated measures ANOVA was conducted on the mental arithmetic condition, with Time and Operator type (addition/subtraction) as independent variables. There was a main effect of Time (*F*(8,152) = 7.345, *p*<0.001,*η*_*p*_^*2*^ = 0.279,*ε* = 0.561) and no main effect for Operation (*F*(1,19) = 1.278, p = 0.272) or an interaction between time and operation (*F*(8, 152) = 1.216, *p* = 0.312, *ε* = 0.457). This indicates that there was no difference in the effect of time on saliency between the easier addition trials and the more difficult subtraction trials.

#### Inter-subject similarity

The NSS-similarity during visual exploration (*M* = 3.25, *SD* = 0.66) was higher than during the mental arithmetic task (*M* = 2.53, *SD* = 0.99; *t*(19) = 4.3,*SD* = 0.7 *p*<0.001,cohens'd = 0.8), suggesting that scan-paths of individual observers were more similar to each other when participants were visually motivated. Nonetheless, the NSS-similarity score was significantly above chance (0) even in the mental arithmetic condition (*t*(19) = 11.43, *SD* = 1, *p*<0.001), suggesting that scan-paths were similar among participants even when visual information was completely task irrelevant.

#### Fixation duration

Fixation durations were longer in the mental arithmetic (*M* = 649.32, *SD* = 344.18) than in the visual exploration condition (*M* = 297.66, *SD* = 59.2; *t*(19) = 4.8, *p*<0.001, Cohen's d = 1.24; **Fig 3E and 3F**). There was no difference in fixation duration between the addition (easier) and the subtraction (more difficult) operations (*t*(19) = -0.92, *p* = 0.370).

#### First fixation latency

The average onset latency of first fixations was later in the mental arithmetic than the visual exploration condition (arithmetic: *M* = 598.28, *SD* = 253.59; exploration: *M* = 262.31, *SD* = 35.58; *t*(19) = 5.91, *SD* = 254.4, *p*<0.001, Cohen's d = 1.87). There was no difference in first fixation latencies between the addition (easier) and the subtraction (more difficult) operations (*t*(19) = -0.91, *p* = 0.375).

#### Central fixation bias

The center bias was evident in both conditions of this experiment: first fixations after image onset were consistently positioned around the image center. This center bias was more pronounced in the mental arithmetic than in the visual exploration task (**Fig 3D**). To examine whether this effect may have been enhanced by the initial central fixation, we performed a control experiment in which the initial fixation was positioned at varied peripheral locations (**Control Experiment 1A, S2 File**). Results (described in the Supporting Information) showed that regardless of the initial fixation position at image onset, the first saccades tended to be directed towards the image center, confirming that there is a center bias of first fixation positions even when the initial fixation position is not at the center (**Panel D in S3 and S2 Figs**). All other findings of Experiment 1 were qualitatively replicated in Experiment 1A.

### Experiment 2

In this experiment there was no visual exploration task. We examined fixation patterns during stimulus presentation when participants were either engaged in a continuous arithmetic task or in a short naming task, completed at an early stage of the trial. In both tasks, all visual information required for successful task performance was fully acquired before the presentation of the cells-RDK display, which was irrelevant for performance. Therefore, for the present purpose, both tasks are considered as non-visual tasks.

#### Gaze samples away proportions (GSP)

GSP was higher in the arithmetic task (*M* = 0.093 *SD* = 0.08) than in the naming task (*M* = 0.065, *SD* = 0.046), but this trend was only marginally significant (*t*(19) = 1.948, *p* = 0.067, Cohen's d = 0.422).

#### Estimation of saliency across fixations

**Fig 4:**
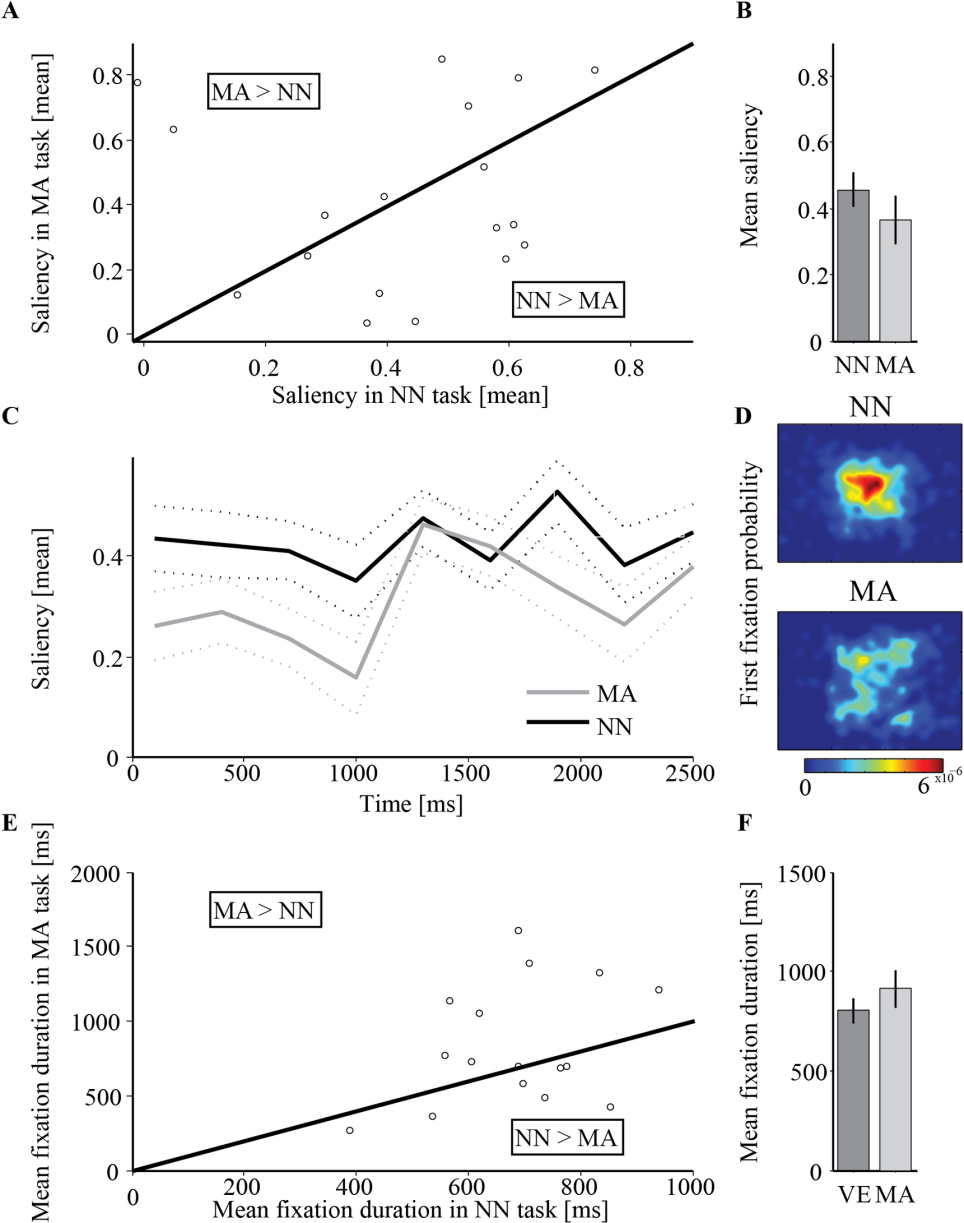
Experiment 2 results. (A) Single-subjects average normalized scan-path saliency (NSS) in the number naming (NN) condition vs. the mental arithmetic (MA) condition. Dots that are below the identity line represent participants for whom the NSS was higher in NN than in MA. (B) Grand average (N = 20) NSS per condition. Error bars denotes ±1 standard error of the mean. (C) Average NSS according to fixations onset times following image presentation (at zero). Dotted line denotes ±1 standard error of the mean. (D) Probability density maps for the spatial distribution of the first fixation occurring after image onset. (E) Single-subjects average fixation duration in the NN and MA conditions. Dots that are above the line represent participants for whom fixation duration was longer in the MA task. (F) Grand average fixation duration per condition. Error bars denotes ±1 standard error of the mean.

Each pixel was given a value of either 1 (contains dynamic motion) or -1 (uniform grey). Due to the structure of the stimuli, this necessarily resulted in an average saliency of 0 for each cell-RDK stimulus. In both tasks, the average saliency was significantly above the expected average saliency of a random observer (Arithmetic: unbiased: *M* = 0.018, *t*(19) = 4.56, *p*<0.001, *Cohen's d* = 1.68; biased: *M* = -0.039, *t*(19) = 4.93,*p*<0.001, *Cohen's d* = 1.52; Naming: unbiased: *M* = 0.018, *t*(19) = 9.9, *p*<0.001, *Cohen's d* = 2.48; biased: *M* = -0.039, *t*(19) = 10.73,*p*<0.001, *Cohen's d* = 2.31). This suggests that participants tended to explore the dynamic pattern even when it was completely task irrelevant and potentially distracting. The average saliency at fixated positions of the number naming condition (*M* = 0.456, *SD* = 0.224) was similar to that of the mental arithmetic condition (*M* = 0.367, *SD* = 0.319; *t* (19) = 0.95, *SD* = 0.42, *p* = 0.352; **Fig 4A and 4B**).

To test the relation between saliency and fixation time we conducted a two-way repeated-measures ANOVA with Time and Viewing-condition, as described for the previous experiments. The main effect of viewing condition was insignificant (*F* (1, 19) = 1.57, *p* = 0.226), which was not surprising considering that both were non-visual tasks. The main effect of time was significant (*F* (8, 152) = 2.31, *p* = 0.023, *η*_*p*_^*2*^ = 0.108), and the interaction between the factors was insignificant (*F* (8, 152) = 0.81, *p* = 0.591, *η*_*p*_^*2*^ = 0.041). Unlike the previous two experiments, the negative linear trend across time was not significant (*F* (1, 19) = 3.071, *p* = 0.096; **Fig 4C**). A separate repeated measures ANOVA was conducted on the mental arithmetic condition, with Time and Operator type (addition/subtraction) as independent variables. There was no main effect of Time (*F*(8,152) = 1.367, *p* = 0.245, *ε* = 0.609), Operation (*F*(1,19) = 1.609, p = 0.220) or an interaction between time and operation (*F*(8,152) = 0.668, *p* = 0.645, *ε* = 0.609)).

#### Fixation duration

We did not expect to find differences between the tasks as they were both non-visual and indeed found none (arithmetic: *M* = 911.56, *SD* = 412.82; naming condition: *M* = 801.71, *SD* = 269.21; *t* (19) = 1.34, *SD* = 367.73, *p* = 0.197; **Fig 4E and 4F**). There was no difference in fixation duration between the addition (easier) and the subtraction (more difficult) operations (*t*(19) = -0.688, *p* = 0.500).

#### First fixation latency

Average onset of first fixations was longer in the mental arithmetic condition (*M* = 674.05, *SD* = 240.72) than the number naming condition (*M* = 544.902, *SD* = 134.57; *t* (19) = 3.51, *SD* = 164.59, *p* = 0.002, Cohen's d = 0.563). There was no difference in first fixation latency between the addition (easier) and the subtraction (more difficult) operations (*t*(19) = 1.095, *p* = 0.287).

#### Central fixation bias

Regardless of the initial fixation at stimulus onset, first saccades tended to be directed towards the image center, resulting in higher probability for the first fixation to be located around the center. This center bias was clearly evident in the naming task, but was less pronounced in the mental arithmetic task (**Fig 4D**).

## Discussion

Comparing the image-scanning patterns during visual (exploration) and non-visual (mental arithmetic) tasks revealed both similarities and differences. During both tasks, participants gazed at the images for most of their presentation time. However, in the non-visual task there was some manifestation of the previously reported gaze-aversion behavior as more time was spent outside the image borders than in the visual task (Experiment 1 and 2). For the main interest of this study we focused only on fixations positions within the image borders.

Consistently with previous findings [50] we found that saliency is highly predictive of fixation locations during visual tasks. The novelty of the present findings is in showing that saliency can be predictive of fixation locations not only in visual tasks but also during non-visual tasks, when the visual input is entirely irrelevant for performance. This was reflected by saliency values which exceeded the average saliency expected by a random observer. Moreover, the pattern of scanning behavior across time was qualitatively similar for the visual and the non-visual tasks: in both cases we observed a reduction in average fixation saliency with the passage of time and a tendency to fixate the center immediately after stimulus onset (“center bias”). Nevertheless, we find quantitative differences between the tasks in scanning-by-saliency behavior: the predictive power of saliency for the non-visual task was reduced relative to the visual task, and this reduction was consistent across time. Additionally, we found that the non-visual task induced slower scanning, as manifested by longer fixations and delayed first saccades in comparison to the visual task.

### Saliency and gaze behavior

We suggested three alternative hypotheses to characterize the link between gaze and saliency in non-visual tasks: a) that gaze is consistently directed away from saliency and focus on less salient locations; b) that gaze is directed to random locations regardless of saliency; c) that gaze is directed toward salient locations similarly to when performing deliberate visual exploration.

We find that bottom-up saliency is predictive of gaze, even when the task is non-visual, compatible with hypothesis c. Saliency was predictive not only for the first fixation (which is highly affected by the center bias, see below), but for fixations occurring during longer durations of viewing, up to the three seconds which we measured. This finding suggests that saliency has a role in the selection of gaze positions regardless of the task. This is especially intriguing in light of the known adversary effects of visual distraction on the performance of non-visual tasks [35,51–54]. Despite these potentially disruptive effects, and despite the easy solution of gazing away from a salient position on the image, human gaze is strongly attracted to visual saliency. This may arguably be a manifestation of a mandatory exploration system which continuously explores the visual surroundings even when attentional resources are busy performing other tasks, consistently with hypothesis C above.

Our findings provide, however, only partial support for hypothesis C. Despite the qualitative equivalence of scanning-by-saliency in both the visual and the non-visual tasks, there was quantitative difference between them: saliency was not as predictive of gaze position in the non-visual task, as it was in the visual task. This indicates that, in addition to this “mandatory” component, the effect of saliency is nevertheless modulated by top-down attentional and motivational goals. When saliency is relevant for the task, as it is in the visual exploration task, its effect on gaze patterns is more substantial than when it is irrelevant. A recent review points at the Midbrain's superior colliculus (SC) as the most likely candidate for handling the neural computations of saliency and overt gaze orientation [55]. According to this view, maps of visual saliency (in the superficial layers of the SC), feed into maps of priority selection maps (in the deeper layers of the SC) that represents top-down goals. Consistently with this model, we propose that non-visual task instructions exert a global non-spatially-specific inhibition of the SC priority map. Since the saliency map remains unchanged but is combined with an inhibited priority map, the outcome is a slowed-down activation. This prediction of this model is a slowed down exploratory-like behavior during non-visual task, as in our findings. We conclude that the findings support a combination of hypothesis B and C, but provides no evidence for hypothesis A.

Consistently with previous findings [22,25,49] we find that the degree by which saliency determines gaze positions is reduced over time. In the visual exploration task (Experiment 1), we found that the average saliency peaked around the onset of the stimulus and gradually reduced for later fixations. This negative relationship between viewing time and average saliency can be explained by a prioritization process, which increases the probability that highly salient positions would be fixated earlier. This temporal modulation of saliency mapping was evident not only in the visual task but also in the non-visual task, providing additional evidence that in the non-visual task the visual system selected fixation targets similarly to selection during visually motivated exploration.

We hypothesized that visual exploration is mandatory and involuntary. However, we cannot rule out the possibility that participants were voluntarily engaged in visual exploration while they were performing the arithmetic task, possibly because they suspected that they will be eventually asked about the images despite being explicitly told otherwise. We believe that this is an unlikely scenario for a few reasons. First, when questioning participants after the experiment, they consistently reported not feeling obliged to look at the images or anywhere else. Second, performance in the rather demanding arithmetic task was around 70%, suggesting that participants were highly engaged in this task. It is unlikely that they were deliberately performing a visual task at the same time. Finally, the hypothesis that participants speculated that they will be later asked about the images is highly unlikely in the second experiment, where the stimuli were simple RDKs with no semantic contents. Still, even in the second experiment we found scanning-by-saliency behavior.

In order to examine the effect of cognitive load on exploratory behavior we compared scanning behavior patterns for mental arithmetic trials of different operations: addition and subtraction. Trials of the subtraction operation were found to be more difficult than trials of the addition operation, as indicated by a lower number of mental operations (space-bar presses) in correct trials and no trade-off with accuracy. Despite the presumed higher cognitive load required by the subtraction trials, there was no difference in exploratory behavior for the two trial types. This finding may indicate that (at least with the tasks we used here) the oculomotor system was automatically engaged in visual exploration, regardless of task difficulty. We consider this finding as preliminary evidence for the automaticity of visual exploration. A more refined control of cognitive load could be achieved by other methods, such as using only one arithmetic operation (e.g. addition) and manipulating the added number [56]. If confirmed in future studies, applying a more refined control on cognitive load, this finding would indicate that visual exploration does not depend on the availability of attentional resources.

### Gaze aversions: Large-scale vs. small-scale

In both the visual and the non-visual tasks, participants spent most of the trial gazing within the image borders. The proportion of time spent outside the image (large-scale gaze aversions) in the mental arithmetic task was lower than previously reported with a similar task and dynamic visual stimuli [40]. This was the case even in Experiment 2 where stimuli were non-semantic and similar to those used in the previous study. The reason for this is probably the tunnel we used to block outside stimulation which worked as an implicit instruction to gaze toward the screen. This manipulation was successful in reducing the amount of large-scale gaze aversions, which was important for understanding the fine-scale fixation patterns during image scanning. However, even with this relatively small number of gaze aversions, we found the previously-reported tendency to perform more large-scale aversions during non-visual relative to visual tasks.

Saliency can be avoided, in theory, not only by large eye movements away from the screen but also by smaller shifts of the gaze toward less salient locations. High acuity vision in humans is restricted to only a small area around the center of fixation (the fovea), therefore, gazing only a few degrees away from a salient position on an image could reduce the disruptive input substantially. This view is reflected by hypothesis A above, suggesting that during non-visual tasks there would be a negative correlation between saliency and gaze positions. Our data provide, however, no evidence supporting this view. On the contrary, we find evidence for a tendency to fixate more on salient locations. This tendency was demonstrated even for dynamic simple stimuli (Experiment 2). Moreover, the stimuli presented in Experiment 2 were binary in their saliency values (more/less salient), which means that saliency could have been easily avoided by small-scale shifts of gaze to less salient locations. Even in this binary case, we observe the opposite trend of attraction to saliency rather than avoidance. These findings suggest that whereas visual distraction can be avoided on a large scale by large aversions away from the image, it cannot be as easily avoided on a small-scale because gaze is strongly attracted to saliency.

### The central fixation bias

When a new image onsets, observers tend to fixate initially toward its center regardless of its saliency [21,25,26,57,58]. This “central fixation bias” is evident to varying degrees in the experiments of the present study. In Experiment 1, the initial fixation position was forced to be at the center at the mental arithmetic task but not at the visual task, therefore, we trivially find more prominent center bias in the mental arithmetic task. In Experiment 1A (see S2 File) and Experiment 2, the initial fixation position was controlled in all conditions and therefore they were better designed to study the center bias. In Experiment 1A, we find a more prominent center fixation bias for the visual exploration task than the mental arithmetic task. In Experiment 2, we find a prominent center bias in the number-naming condition but lower central bias in the mental arithmetic task.

A few non-mutually-exclusive hypotheses were previously suggested to explain this bias. One of them is that photographers often position the most interesting item in the center of their photographs, making the image center an informative (and salient) region of the image. In this case, the center bias is partially caused by presenting images taken by photographers. This photographer bias may also influence viewing strategies indirectly through an acquired scanning bias, developed by previous experience with this common artificial layout of images [26,59]. A more general hypothesis is that fixating the center first provides an optimal location from which to subsequently explore the scene [57]. According to these views, whereas the center bias is not strictly tuned to visual saliency, it still serves as a useful strategy for the acquisition of visual information, as it positions the gaze at a potentially salient region which is also an optimal position for further exploration.

In the mental arithmetic task there was a lower motivation for exploration than in the visual exploration task of Experiment 1 or the naming task of Experiment 2, in which there was effectively no task during stimulus presentation. These differences in motivation may explain the differences we observed between conditions in their center bias. First, less motivation may attenuate the tendency to employ optimal viewing strategies such as the center bias. Second, less motivation may induce slower scanning patterns and result, among other effects, in a later first fixation, which was recently shown to relate to a less pronounced center bias [58].

Notably, in Experiment 1 and 1A, using natural images, the central bias was still robust even in the mental arithmetic task, indicating that attentional task demands are not enough to override the bias in this case. In Experiment 2, with the artificial simple stimuli, we found reduced center bias. This is probably due to the different stimulus properties: it contained no photographer bias, no semantic information and might have been perceived as multiple objects rather than a single image (due to its cell structure). We conclude that, similarly to other examined viewing strategies, the center bias is evident (although attenuated) even when the task demands do not exert exploratory motivation.

### Free-view vs. non-sensory task

Three tasks are typically employed in saliency mapping studies: free-view exploration [15,25–29], scene memorization [19–21] and visual search [19,22–24]. Free-view was traditionally used to examine scanning behavior that is free of top-down influences, as it is assumed that subjective motivations and intentions vary and are thus canceled when averaged across participants. This task-independent mode of viewing is considered the "default" state of the visual system. Recently, however, the appropriateness of the free-view procedure in studying this default state was questioned [15]. It was suggested that under free view instructions, participants tend to be engaged in visual tasks such as scene memorization and categorization. According to this view, the free-view procedure is not “task-independent” as previously suggested but it is highly affected by the motivation to acquire visual information and act upon it, just as the memorization and search task are. In contrast, in real life, there is not always high motivation for visual exploration, as we are often exposed to visual information which is entirely irrelevant for our purposes and goals.

We suggest that a non-sensory task (such as mental arithmetic) can complement the free-view procedure for examining task-independent viewing behavior. Such tasks imitate real life, where humans are engaged in internal cognitive processes while being exposed to visual information. Non-visual tasks minimize visually-motivated top-down effects, by incorporating alternative non-visual motivations. These motivations are not “task-independent” as they are affected by a non-visual task, but they are not driven by visual goals, and can therefore better represent the “default” state of visual scanning.

## Conclusion

Fine-scale aversion-from-saliency was not evident during a non-visual mental arithmetic task. Instead, exploratory mechanisms such as the center bias and saliency “maximization” are manifested in the non-visual task, where they are entirely task irrelevant. This may be taken as evidence that exploratory scanning behavior has a “mandatory” component which makes it partially unavoidable. Even when this scanning is useless for task performance, it is still performed to a certain extent. However, we find that there are quantitative differences in the manifestation of exploratory mechanisms in the two tasks: in the non-visual task fixations are longer and the first fixation starts later than in the visual task. We interpret these quantitative differences as reflecting the lower motivation for exploration in the non-visual than the visual task. We conclude that during non-visual tasks, the visual system samples the visual information at a lower rate but based on similar target selection mechanisms as those that govern visually motivated tasks.

## Supporting information

S1 File Analysis using Itti et al.’s algorithm. (DOCX)

S2 File Methods and results of Control Experiment 1A. (DOCX)

S1 Fig Experiment 1 NSS results. (A) Single-subjects average normalized scan-path saliency (NSS) in the visual exploration (VE) condition vs. the mental arithmetic (MA) condition. Dots that are below the identity line represent participants for whom the NSS was higher in VE than in MA. (B) Grand average (N = 20) NSS per condition. Error bars denotes ±1 standard error of the mean. (C) Average NSS according to fixations onset times following image presentation (at zero). Dotted line denotes ±1 standard error of the mean.
(TIF)

S2 Fig Experiment 1A NSS results. (A) Single-subjects average normalized scan-path saliency (NSS) in the visual exploration (VE) condition vs. the mental arithmetic (MA) condition. Dots that are below the identity line represent participants for whom the NSS was higher in VE than in MA. (B) Grand average (N = 20) NSS per condition. Error bars denotes ±1 standard error of the mean. (C) Average NSS according to fixations onset times following image presentation (at zero). Dotted line denotes ±1 standard error of the mean.
(TIF)

S3 Fig Experiment 1A results. (A) Single-subjects average normalized scan-path saliency (NSS) in the visual exploration (VE) condition vs. the mental arithmetic (MA) condition. Dots that are below the identity line represent participants for whom the NSS was higher in VE than in MA. (B) Grand average (N = 20) NSS per condition. Error bars denotes ±1 standard error of the mean. (C) Average NSS according to fixations onset times following image presentation (at zero). Dotted line denotes ±1 standard error of the mean. (D) Probability density maps for the spatial distribution of the first fixation occurring after image onset. (E) Single-subjects average fixation duration in the VE and MA conditions. Dots that are above the line represent participants for whom fixation duration was longer in the MA task. (F) Grand average fixation duration per condition. Error bars denotes ±1 standard error of the mean.
(TIF)
