# Supplementary material for: Oculomotor behavior during non-visual tasks: The role of visual saliency
Source: PLoS One. 2018 Jun 22;13(6):e0198242. doi: 10.1371/journal.pone.0198242 (PMC6014668; doi:10.1371/journal.pone.0198242)
Supplement: S1 File — (DOCX) [file pone.0198242.s001.docx]

**S1: Analysis using Itti et al.’s algorithm**

Saliency was computed using the Matlab saliency toolbox (Walther & Koch, 2006) using the prevailing bottom-up saliency model of Itti, Koch and Niebur (1998). This was done to generalize our results over different saliency algorithm types.

**Experiment 1:**

**Estimation of saliency across fixations**

The average saliency in the VE condition was significantly higher than in the MA task *t*(19)=5.036, *p*<0.001, *Cohen's d*=1.6867, **Panel B in** **Fig S1**. Nevertheless, saliency in the MA condition was significantly higher than a simulated random observer (Unbiased: *t*(19)=11.165, *p*<0.001, *Cohen's d*=3.3505; Biased: *t*(19)=6.055,*p*<0.001, *Cohen's d*=1.9304). This suggests that saliency was a better-than-chance predictor for gaze positions even when the visual information was irrelevant. To test the relation between saliency and fixation time we conducted a two-way repeated-measures ANOVA with Time and Viewing-condition as described in Experiment 1. A significant main effect was found for viewing-condition (*F*(1,19)=29.786,*p*<0.001, *ɳ_p_^2^*=0.611) and time (*F*(8,152)=7.652, *p*<0.001, *ɳ_p_^2^*=0.287, *ε*=0.524). The interaction between time and condition was also only marginally significant (*F*(8,152)=2.252, *p*=0.084, *ε*=0.419). When analyzing the two conditions separately, we found significant negative linear trends of time in both conditions, but the trend was stronger in the visual exploration condition (arithmetic: F(1,19)=11.923,p=0.003, *ɳ_p_^2^*=0.386; exploration: *F*(1,19)=88.876,*p*<0.001, *ɳ_p_^2^*=0.824; Panel C in **Fig S1**). A separate repeated measures ANOVA was conducted on the mental arithmetic condition, with Time and Operator type (addition/subtraction) as independent variables. There was a main effect of Time (*F*(8,152)=2.962, *p*=0.018, *ɳ_p_^2^*=0.135, *ε*=0.586) and no main effect for Operation (*F*(1,19)=2.620, *p*=0.122) or an interaction between time and operation (*F*(8,152)=1.206,*p*=0.312, *ε*=0.619).

These results suggest that visual saliency plays a role in determining gaze target selection and generalize the results of our main analysis to a different type of saliency algorithm.

**Fig S1.** **Experiment 1 NSS results.** (A) Single-subjects average normalized scan-path saliency (NSS) in the visual exploration (VE) condition vs. the mental arithmetic (MA) condition. Dots that are below the identity line represent participants for whom the NSS was higher in VE than in MA. (B) Grand average (N=20) NSS per condition. Error bars denotes ±1 standard error of the mean. (C) Average NSS according to fixations onset times following image presentation (at zero). Dotted line denotes ±1 standard error of the mean.

**Control Experiment 1A:**

**Estimation of saliency across fixations**

The average saliency in the VE condition was significantly higher than in the MA task *t*(19)=5.907, *p*<0.001, *Cohen's d*= 1.92, **Panel B in** **Fig S2**. Nevertheless, saliency in the MA condition was significantly higher than a simulated random observer (Unbiased: *t*(19)=10.297, *p*<0.001, *Cohen's d*=3.4197; Biased: *t*(19)=5.658,*p*<0.001, *Cohen's d*=1.612). This suggests that saliency was a better-than-chance predictor for gaze positions even when the visual information was irrelevant. To test the relation between saliency and fixation time we conducted a two-way repeated-measures ANOVA with Time and Viewing-condition as described in Experiment 1. A significant main effect was found for viewing-condition *F*(1,19)=32.475,*p*<0.001, *ɳ_p_^2^*=0.596) and time (*F*(8,152)=14.2, *p*<0.001, *ɳ_p_^2^*=0.428, *ε*=0.541). The interaction between time and condition was also significant *(F*(8,152)=2.697, *p*=0.037, *ɳ_p_^2^*=0.063, *ε*=0.496). When analyzing the two conditions separately, we found significant negative linear trends of time in both conditions, but the trend was stronger in the visual exploration condition (arithmetic: F(1,19)=13.349,p=0.002, *ɳ_p_^2^*=0.413; exploration: F(1,19)=107.452,p<0.001, *ɳ_p_^2^*=0.850; **Panel C in Fig S2**). A separate repeated measures ANOVA was conducted on the mental arithmetic condition, with Time and Operator type (addition/subtraction) as independent variables. There was a main effect of Time (*F*(8,152)=5.753, *p*<0.001, *ɳ_p_^2^*=0.232, *ε*=0.588) and no main effect for Operation (*F*(1,19)=0.651, *p*=0.430)) or an interaction between time and operation (*F*(8,152)=1.164,*p*=0.324).

These results suggest that visual saliency plays a role in determining gaze target selection and generalize the results of our main analysis to a different type of saliency algorithm.

**Fig S2.** **Experiment 1A NSS results**. (A) Single-subjects average normalized scan-path saliency (NSS) in the visual exploration (VE) condition vs. the mental arithmetic (MA) condition. Dots that are below the identity line represent participants for whom the NSS was higher in VE than in MA. (B) Grand average (N=20) NSS per condition. Error bars denotes ±1 standard error of the mean. (C) Average NSS according to fixations onset times following image presentation (at zero). Dotted line denotes ±1 standard error of the mean.

**References:**

Itti, L., Koch, C., & Niebur, E. (1998). A model of saliency-based visual attention for rapid scene analysis. *IEEE Transactions on Pattern Analysis and Machine Intelligence*, *20*(11), 1254–1259.

Walther, D., & Koch, C. (2006). Modeling attention to salient proto-objects. *Neural Networks*, *19*(9), 1395–1407.
