# Supplementary material for: Oculomotor behavior during non-visual tasks: The role of visual saliency
Source: PLoS One. 2018 Jun 22;13(6):e0198242. doi: 10.1371/journal.pone.0198242 (PMC6014668; doi:10.1371/journal.pone.0198242)
Supplement: S2 File — (DOCX) [file pone.0198242.s002.docx]

**S2: Control Experiment 1A**

In Experiment 1 we examined visual exploration during a demanding non-visual task and revealed the critical role of saliency and the center-bias in determining fixations on a task-irrelevant visual image. However, in that experiment the initial fixation was always located in the center when the image was presented. This could have caused a bias to fixate at the center and could also artificially increase saliency measurements because the center tends to be more salient in many images. In Control Experiment 1A we examined the same effects but with non-central locations of the initial fixation. This experiment was almost identical to Experiment 1, except that pre-stimulus fixation was located at varied peripheral locations other than the center.

**Methods**

**Participants**

This experiment included 20 participants (9 females, Mean age = 24.45, Range 19-35, SD = 4.01). All participants reported being healthy, with no history of neurological disorders and with normal or corrected-to-normal vision and normal hearing. The experiment has gone through the same ethical procedure as Experiment 1.

**Apparatus**

Same as in Experiment 1.

**Stimuli**

Same as Experiment 1, except that three images were added to each stimulus set.

**Procedure**

Experiment 2 was almost identical to Experiment 1, except that participants started each trial by fixating the periphery rather than the center of the screen. In the mental arithmetic task, the initial number and the operator were presented in one of four peripheral positions (up-right, up-left, down-right, down-left), at a distance of 6^o^ from the center. As in Experiment 1, no explicit instruction was given as to the location of gaze. Participants naturally looked at the peripheral locations, to be able to perform the following arithmetic task. In the visual exploration blocks, the blank screen of Experiment 1 was replaced by the presentation of a fixation cross in one of the same four peripheral positions. Participants were instructed to fixate the cross at the beginning of each trial and the image was presented only after they did so continuously for 1 second.

In this experiment, there were slightly more trials than in Experiment 1: each experimental block included 16 trials (48 trials in total). This was done so that the number of trials would be divided by 4 to balance the 4 options of initial fixation positions. Also, there were 48 possible "starting numbers" used for all the participants in a random order.

**Results**

**Task compliance and behavioral performance**

Accuracy-rates of the mental arithmetic task were higher than chance (*M*=0.75, *SD*=0.1, Range 0.59-0.92). On average, participants performed more than 2 operations in correct trials (*M*=2.52, *SD*=0.79, Range 1.4-3.8). There were significantly more mental operations in correct addition than subtraction trials (*Addition*: *M*=2.65, *SD*=0.87; *Subtraction*: *M*=2.44, *SD* =0.81; *t*(19)=2.597, *p*=0.018, Cohen's d=0.257). This effect cannot be explained by a trade-off between number of successful mental operations and accuracy because accuracy in addition trials was either equal to or higher than accuracy in subtraction trials (Accuracy rates: *Addition*: *M*=0.75, *SD* =0.11; *Subtraction*: *M*=0.75, *SD*=0.12; *t*(19)=0.285, *p*=0.78). This indicates that the subtraction task was more difficult than the additional task. Accuracy-rates of the memory test (classifying new/old) performed after the exploration blocks were above chance (0.5) for all participants (*M*=0.9, *SD*=0.085).

**Gaze samples away proportions (GSP)**

GSP was higher in the arithmetic task (*M*=0.131, *SD*=0.127) than in the exploration task (*M*=0.021, *SD*=0.017; *t*(19)=3.97, *p*<0.001, Cohen's d=1.04).

**Estimation of saliency across fixations**

The average NSS in both the visual exploration and the mental arithmetic condition was significantly above the expected average NSS of a random observer (Arithmetic: unbiased: *M*=-0.005, *t*(19)=9.396, *p*<0.001, *Cohen's d*=2.54; biased: *M*=0.447, *t*(19)=7.403, *p*<0.001, *Cohen's d*=2.06; Exploration: unbiased: *M*=-0.005, *t*(19)=32.65, *p*<0.001, *Cohen's d*=10.58; biased: *M*=0.447, *t*(19)=31.24, *p*<0.001, *Cohen's d*=7.21), suggesting that, as in Experiment 1, saliency played a crucial role in determining gaze position even in the non-visual task. Nevertheless, the average saliency at fixated positions during the visual exploration condition (*M*=3.693, *SD*=0.495) was significantly higher than the average saliency at fixated positions during the mental arithmetic condition (*M*=1.957, *SD*=0.930; *t*(19)=8.08, *SD*=0.96, *p*<0.001, Cohen's d=2.34; **Panels A & B in** **Fig S3**). This indicates that saliency was more influential on gaze position in the visual than the non-visual task.

To test the relation between saliency and fixation time we conducted a two-way repeated-measures ANOVA with Time and Viewing-condition as described in Experiment 1. A significant main effect was found for viewing-condition (*F*(1, 14)=55.55, *p*<0.001, *ɳ_p_^2^*=0.75) and time (*F*(8, 152) =28.101, *p*<0.001, *ɳ_p_^2^*=0.6). The interaction between time and condition was also significant (*F*(8,152)=3.2, *p*=0.002, *ɳ_p_^2^*=0.144, *ε*=0.587). When analyzing the two conditions separately, we found significant negative linear trends of time in both conditions, but the trend was stronger in the visual exploration condition (arithmetic: *F*(1, 14)=17.62, *p*<0.001, *ɳ_p_^2^*=0.481; exploration: *F*(1, 14)=208.28, *p*<0.001, *ɳ_p_^2^*=0.916; **Panel C in Fig S3**). A separate repeated measures ANOVA was conducted on the mental arithmetic condition, with Time and Operator type (addition/subtraction) as independent variables. There was a main effect of Time (*F*(8,152)=5.197, *p*<0.001, *ɳ_p_^2^*=0.215) and no main effect for Operation (*F*(1,19)=2.238, *p*=0.151 ) or an interaction between time and operation (*F*(8,152)=0.645, *p*=739 ).

**Inter-subject similarity**

The NSS-similarity during visual exploration (*M*=3.55, *SD*=0.39) was higher than during the mental arithmetic (*M*=1.98, *SD*=0.72) task (*t*(19)=10.39,*SD*=0.68 *p*<0.001,cohens'd=2.599) suggesting that scan-paths of individual observers were more similar to each other when participants were visually motivated. Nonetheless, the NSS-similarity score was significantly above chance (0) even in the mental arithmetic condition (*t*(19)=12.294, *SD*=0.72, *p*<0.001), suggesting that scan-paths shared significant similarities even when visual information was completely task irrelevant.

**Fixation duration**

Fixation durations were longer in the mental arithmetic (*M*=527.64, *SD*=295.19) than in the visual exploration condition (*M* =299.061, *SD*= 44.547; *t* (19) =3.367, *SD*=303.599, p=0.003, Cohen's d=0.668; **Panel E & F in Fig S3**). There was no difference in fixation duration between the addition (easier) and the subtraction (more difficult) operations (*t*(19)=-0.467, *p*=0.646).

**First fixation latency**

The average onset latency of first fixation was later in the mental arithmetic than in the visual exploration condition (arithmetic: *M*=458.39, *SD*=201.16; exploration: *M*=258.053, *SD*=37.198; *t* (19) =4.38, *SD*=204.39, *p*<0.001, Cohen's d=1.38). There was no difference in first fixation latencies between the addition (easier) and the subtraction (more difficult) operations (*t*(19)=-1.976, *p*=0.063).

**Central fixation bias**

Regardless of the initial fixation position at image onset, the first saccades tended to be directed towards the image center, confirming that there is a center bias of first fixation positions even when the initial fixation position is not at the center (**Panel D in Fig S3**). In this case we find more pronounced center bias in the visual exploration than in the mental arithmetic condition.


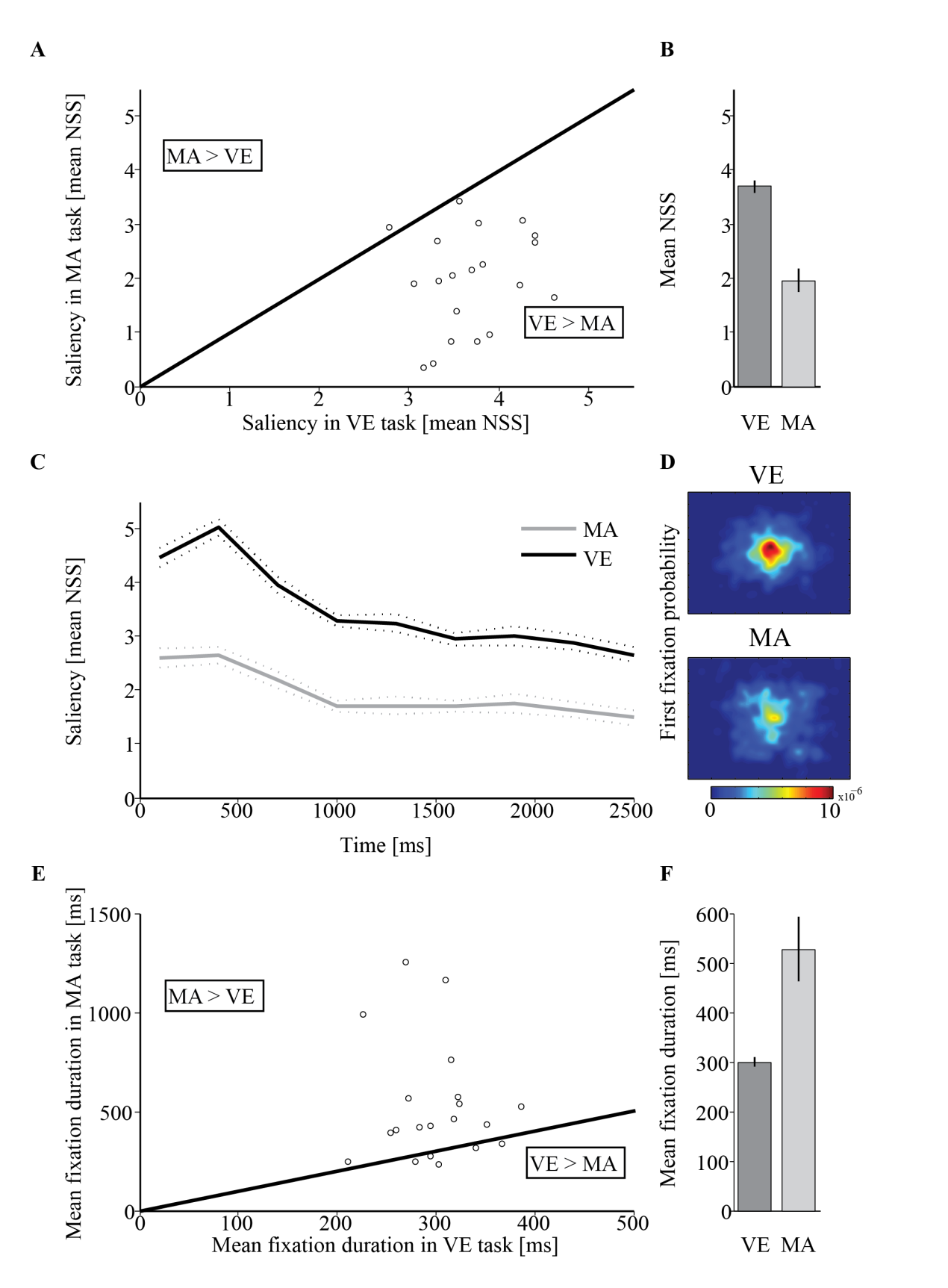


**Fig S3.** **Experiment 1A results.** (A) Single-subjects average normalized scan-path saliency (NSS) in the visual exploration (VE) condition vs. the mental arithmetic (MA) condition. Dots that are below the identity line represent participants for whom the NSS was higher in VE than in MA. (B) Grand average (N=20) NSS per condition. Error bars denotes ±1 standard error of the mean. (C) Average NSS according to fixations onset times following image presentation (at zero). Dotted line denotes ±1 standard error of the mean. (D) Probability density maps for the spatial distribution of the first fixation occurring after image onset. (E) Single-subjects average fixation duration in the VE and MA conditions. Dots that are above the line represent participants for whom fixation duration was longer in the MA task. (F) Grand average fixation duration per condition. Error bars denotes ±1 standard error of the mean.
