# Supplementary figures and images for: Oculomotor behavior during non-visual tasks: The role of visual saliency
Source: PLoS One. 2018 Jun 22;13(6):e0198242. doi: 10.1371/journal.pone.0198242 (PMC6014668; doi:10.1371/journal.pone.0198242)

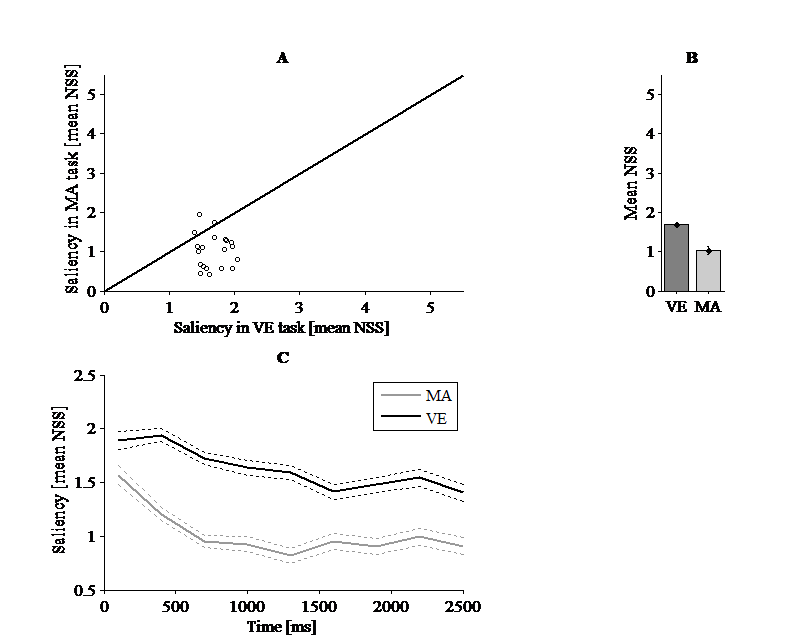

Supplement: S1 Fig — (A) Single-subjects average normalized scan-path saliency (NSS) in the visual exploration (VE) condition vs. the mental arithmetic (MA) condition. Dots that are below the identity line represent participants for whom the NSS was higher in VE than in MA. (B) Grand average (N = 20) NSS per condition. Error bars denotes ±1 standard error of the mean. (C) Average NSS according to fixations onset times following image presentation (at zero). Dotted line denotes ±1 standard error of the mean. (TIF) [file pone.0198242.s003.tif]

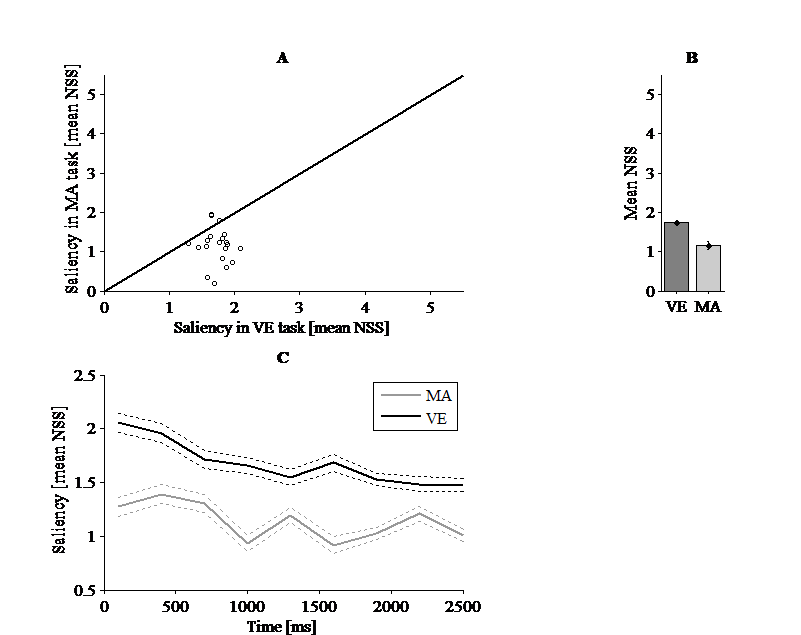

Supplement: S2 Fig — (A) Single-subjects average normalized scan-path saliency (NSS) in the visual exploration (VE) condition vs. the mental arithmetic (MA) condition. Dots that are below the identity line represent participants for whom the NSS was higher in VE than in MA. (B) Grand average (N = 20) NSS per condition. Error bars denotes ±1 standard error of the mean. (C) Average NSS according to fixations onset times following image presentation (at zero). Dotted line denotes ±1 standard error of the mean. (TIF) [file pone.0198242.s004.tif]

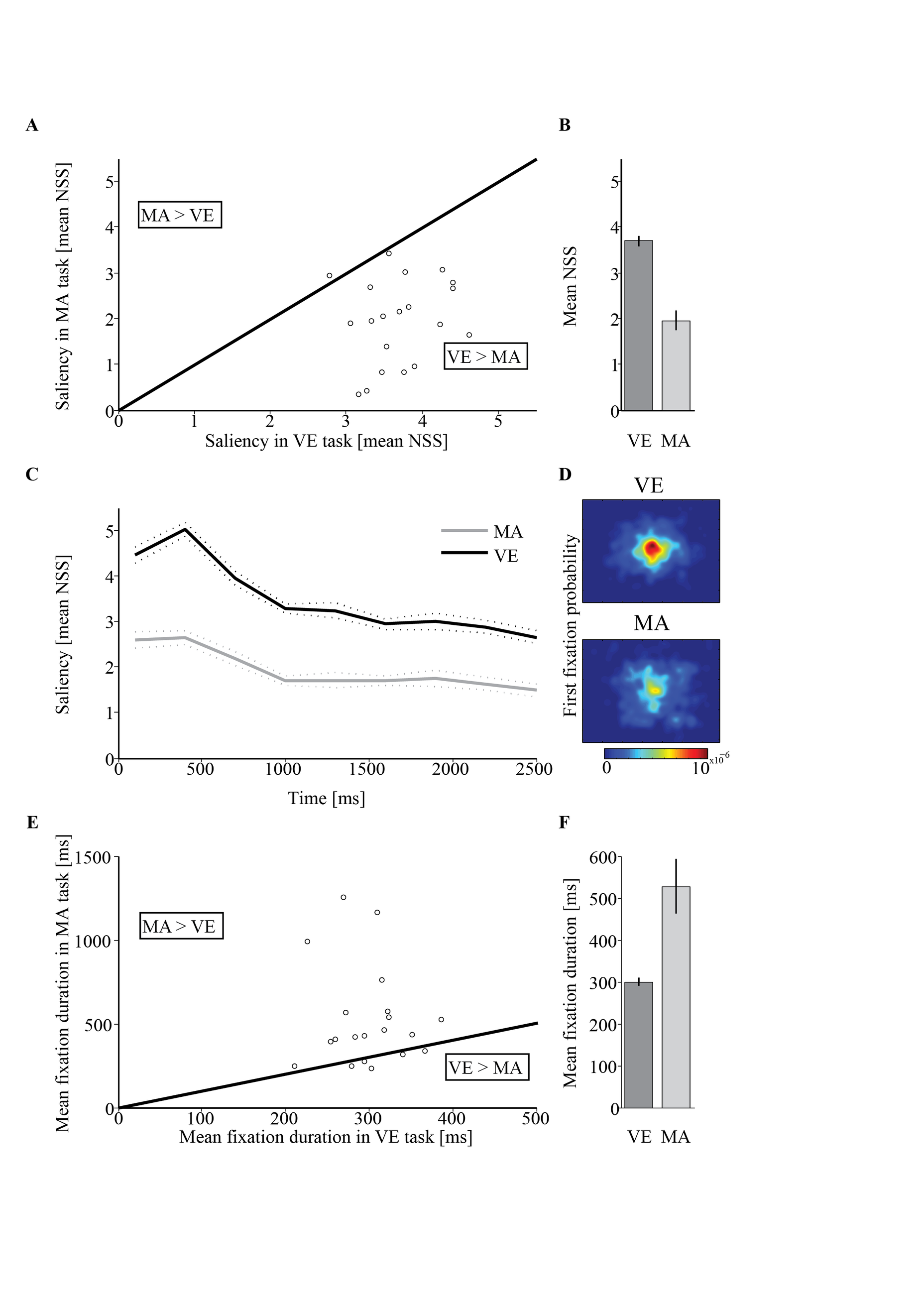

Supplement: S3 Fig — (A) Single-subjects average normalized scan-path saliency (NSS) in the visual exploration (VE) condition vs. the mental arithmetic (MA) condition. Dots that are below the identity line represent participants for whom the NSS was higher in VE than in MA. (B) Grand average (N = 20) NSS per condition. Error bars denotes ±1 standard error of the mean. (C) Average NSS according to fixations onset times following image presentation (at zero). Dotted line denotes ±1 standard error of the mean. (D) Probability density maps for the spatial distribution of the first fixation occurring after image onset. (E) Single-subjects average fixation duration in the VE and MA conditions. Dots that are above the line represent participants for whom fixation duration was longer in the MA task. (F) Grand average fixation duration per condition. Error bars denotes ±1 standard error of the mean. (TIF) [file pone.0198242.s005.tif]
